# Fitness and Morphology Support Genetic Differentiation Across Different Geographic Scales in a Native Insect Utilising Native vs. Invasive Host Plants

**DOI:** 10.1002/ece3.71373

**Published:** 2025-05-12

**Authors:** Johannes J. Le Roux, Levi Brown, Scott P. Carroll, Jessica A. O'Hare, Jess M. Herbert, Niah M. Delamotte, Nicholas Bersee, Sigrid Iredell, Rowan M. Clarke, Selina Kosak, Rachael Y. Dudaniec, Dylan M. Geraghty

**Affiliations:** ^1^ School of Natural Sciences Macquarie University Sydney New South Wales Australia; ^2^ Department of Nematology and Entomology University of California Davis California USA

**Keywords:** hybridisation, invasive species, plant‐insect interactions, rapid evolution

## Abstract

Native species can evolve rapidly in response to utilising invasive species as novel resources. We investigated the genetic and trait differentiation of the Australian soapberry bug *Leptocoris tagalicus* across three biotypes: those feeding on invasive *Cardiospermum grandiflorum* in New South Wales (NSW) and Queensland (Qld), invasive *C. halicacabum* in the Northern Territory (NT), and on the native host *Alectryon tomentosus* (in Qld). Genetic analyses revealed moderate differentiation between NT insects and those from NSW and Qld (*F*_ST_ = 0.033). Conversely, insects from NSW and Qld had low genetic differentiation, irrespective of their host plant associations (*F*_ST_ = 0.008). Field data and data from a multi‐generation experiment indicated ongoing adaptation in proboscis length in insects feeding on the two invasive host plant species, likely in response to the sizes of their fruits. Multi‐generation hybridisation experiments demonstrated high narrow sense heritability in insect proboscis length and body size (H_2_ = 0.48 and 0.4, respectively). Crosses involving F_1_ hybrids of insect biotypes generally outperformed inter‐biotype and control crosses. Taken together, these findings support ongoing genetic differentiation among *L. tagalicus* biotypes across different spatial scales, even in instances of high gene flow.

## Graphical abstract

Native species can rapidly evolve when utilising invasive species as novel resources. We found that the Australian soapberry bug *Leptocoris tagalicus* exhibits genetic differentiation and morphological adaptation across biotypes feeding on different native and invasive host plants. Hybridisation experiments revealed high heritability of key insect traits, supporting ongoing evolutionary divergence despite gene flow.

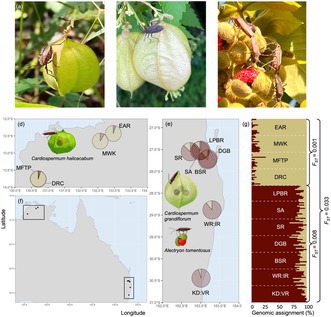

## 1 Introduction

Invasive alien species are significant drivers of global change, impacting biodiversity across all levels of organisation, from genes to ecosystems (IPBES 2023). While invasive species often outcompete and displace native species, some natives may co‐exist with them. Native species may tolerate, evade, or utilise invasives through behavioural plasticity (Ghalambor et al. 2007) or rapid evolutionary responses (Carroll 2011). For example, in response to consuming toxic invasive cane toads ( *Rhinella marinus* ), some Australian snake species evolved smaller heads, reducing their gape sizes to limit predation of larger cane toads that are more toxic than smaller ones (Phillips and Shine 2004).

The strength of the selection imposed by invasive species on native species depends partly on their shared eco‐evolutionary experience (EEE), which pertains to adaptations to biotic interactions over evolutionary timescales (Saul et al. 2013). In the context of biological invasions, EEE relates to the role of pre‐adaptations in determining the ease by which new interactions are formed between natives and invaders, that is, ecological integration into novel communities (Saul et al. 2013; Saul and Jeschke 2015). Invasive species will exert strong selection on native species that they frequently interact with but that lack EEE with them, such as the cane toad and snake example mentioned above. A case of high EEE shared between interacting native and invasive species involves soapberry bugs (true bugs in the genera *Boisea*, *Jadera* and *Leptocoris*), which are obligate seed predators of species in the soapberry family Sapindaceae. Soapberry bugs have colonised invasive soapberry plants in many parts of the world (Carroll and Loye 2012). In some instances, these new associations have led to rapid evolutionary responses in the insects, often involving changes in proboscis length to align with host fruit size for seed access (e.g., Carroll et al. 2005a; Foster et al. 2019).

In Australia, the soapberry bug *Leptocoris tagalicus* has two subspecies, subsp. *tagalicus* and subsp. *vulgaris* (Gross 1960). *Leptocoris tagalicus* subsp. *tagalicus* is larger‐bodied, occurring in eastern and northern wet and dry rainforests, while *L. tagalicus* subsp. *vulgaris* is smaller‐bodied and typically inhabits central desert and northern savanna areas. Both *L. tagalicus* subspecies have colonised invasive *Cardiospermum* balloon vine species in Australia as new food resources (Carroll and Loye 2012). These vines are native to the tropical and warm temperate New World and have evolved large inflated spherical seed capsules (balloons) as an elaborated seed defence system against the piercing sucking mouthparts of soapberry bugs and other insects native there. This mechanical defence system is absent from all native Australian Sapindaceae species and so represents a novel challenge to feeding by native Australian soapberry bugs.

*Cardiospermum halicacabum* was likely introduced into the northern parts of Australia's Northern Territory (NT) between 1600 and 1800 (Bean 2007; Le Roux et al. 2025) and was colonised by both *L. tagalicus* subspecies (Andres et al. 2013). This led to the establishment of a highly differentiated hybrid *L. tagalicus* biotype (hereafter referred to as “halicacabum” insects). While “halicacabum” insects remained small‐bodied, their proboscides increased by as much as 38% (Andres et al. 2013). This rapid evolutionary response allowed them to more efficiently reach seeds inside the inflated balloons of *C. halicacabum* (Andres et al. 2013).

*Cardiospermum grandiflorum* was first recorded in Australia in 1923 as a botanical specimen of a horticultural escape (Carroll et al. 2005b). Particularly since the 1970s, it has invaded large areas along the country's east coast in New South Wales (NSW) and Queensland (Qld) (Carroll et al. 2005b; Le Roux et al. 2025). This balloon vine was colonised by *L. tagalicus* subsp. *tagalicus*. *Cardiospermum grandiflorum* has larger fruits than *C. halicacabum* , and by 2004 field measurements showed that the proboscides of insects feeding on *C. grandiflorum* were around 10% longer than those of insects found on the neighbouring native host plant, *Alectryon tomentosus* (Carroll et al. 2005a). Insects found in association with these two host species are hereafter referred to as “grandiflorum” and “tomentosus” insects, respectively. The independent colonisation of two invasive balloon vine species by *L. tagalicus* in Australia, and the similar evolutionary responses in the insect under different demographic scenarios and temporal scales, provide valuable opportunities to further study the evolutionary impacts of balloon vines on *L. tagalicus*.

The evolutionary trajectories and geographic isolation of “halicacabum” and “grandiflorum” insects in Australia may ultimately lead to reproductive isolation between populations found on different host plants (e.g., Schwarz et al. 2005). Reproductive isolation is expected to evolve more rapidly when gene flow between populations is limited, allowing genomic incompatibilities to accrue more rapidly over time than under conditions of high gene flow. However, theory also suggests that adaptive differentiation can occur between nearby populations with ongoing gene flow, particularly when selection acts on a small number of genes with major effects (Gavrilets and Vose 2005). Although the role of fitness‐related loci in wild populations is still not fully understood (Enbody et al. 2023), evidence indicates that the early stages of speciation often involve a few genomic regions that retain differentiation despite gene flow (Riesch et al. 2017). For instance, a study on the North American soapberry bug, *Jadera haematoloma* , suggested that selection on a limited number of loci may drive trait divergence between insect biotypes adapted to native and non‐native host plants (Carroll et al. 1997).

We aim to determine whether “grandiflorum”, “halicacabum” and “tomentosus” biotypes differ genetically from one another by inferring the genetic structure and comparing the fitness of different crosses between them. We hypothesise that, given the differences in residence time of the two *Cardiospermum* host plant species, together with their geographic isolation in Australia, “halicacabum” insects will be more genetically differentiated from “grandiflorum” and “tomentosus” insects than the latter two will be from each other. We also hypothesise that the greater genetic differentiation between “grandiflorum” and “halicacabum” insects will correspond to significant differences in the fitness of their hybrids compared to that of both parental biotypes, but that experimental hybrids between “grandiflorum” and “tomentosus” insects will have similar fitness to parental biotypes due to their recent divergence and largely overlapping distributions.

## 2 Material and Methods

### 2.1 Field Sampling

**FIGURE 1:**
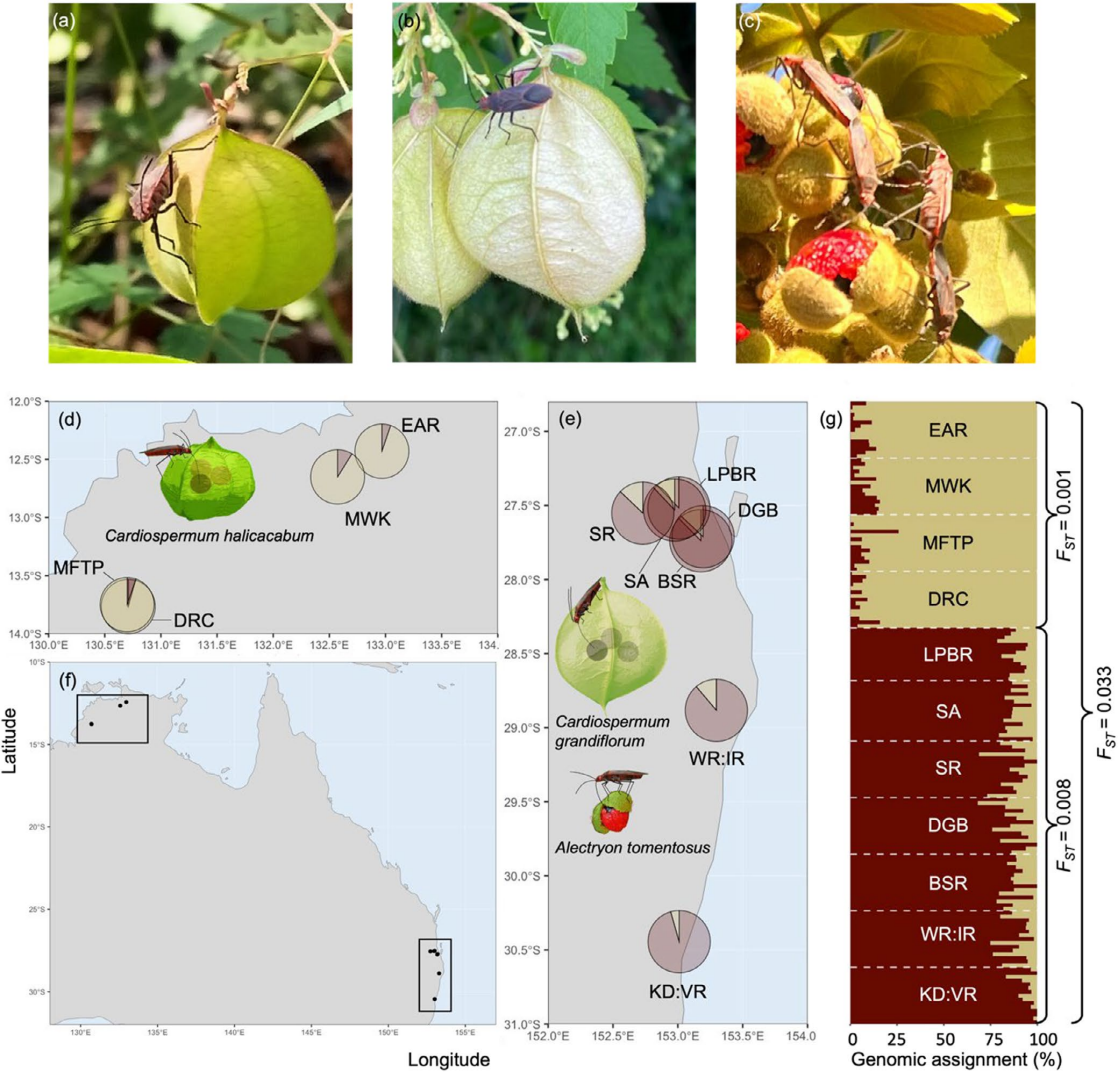
*Leptocoris tagalicus* pictured on two of its invasive host plant species, (a) *Cardiospermum halicacabum,* (b) *C. grandiflorum,* and (c) one of its native host plants *Alectryon tomentosus*. Included are maps illustrating the locations of collection sites of *Leptocoris tagalicus* on (d) *C. halicacabum* in the Northern Territory and (e) *C. grandiflorum* and *Alectryon tomentosus* in the States of New South Wales and Queensland. These insert maps correspond to the rectangles on the larger map (f). Pie charts in insert maps show the average genomic assignment values of insects at each collection site to one of two population genetic clusters. (g) Genetic structure of *L. tagalicus* inferred from ancestral admixture analysis. The vertical bar plot shows the proportion of each individual insect's genomic assignment (*y*‐axis) to each cluster. Average site pairwise *F*_*ST*_ values within and between the two main population genetic clusters are shown. *Leptocoris tagalicus* individuals collected on *C. halicacabum* in the Northern Territory had genomic assignment values corresponding mostly to one cluster (tan colour) while insects collected on *A. tomentosus* and *C. grandiflorum* in New South Wales and Queensland were primarily assigned to a second cluster (maroon colour). Photo credits: Dylan Geraghty (pane a), Johannes Le Roux (pane b) and Levi Brown (pane c).

*Alectryon tomentosus* is one of the main host plants of the *L. tagalicus* subsp. *tagalicus* insects that have colonised both balloon vine species in Australia (Carroll et al. 2005a; Andres et al. 2013). We collected *L. tagalicus* subsp. *tagalicus* from this host plant (i.e., “tomentosus” insects) from two sites: Sherwood Arboretum (SA; Qld) and Bahr Scrub Road (BSR; Qld). Putative hybrids between *L. tagalicus* subsp. *tagalicus* and subsp. *vulgaris* associated with *C. halicacabum* in the NT (i.e., “halicacabum” insects) were collected from four sites: Daly River Crossing (DRC), East Alligator River boat ramp (EAR), Mango Farm Tourist Park (MFTP), and Mamukala Wetland (MKW). *Leptocoris tagalicus* subsp. *tagalicus* was collected from *C. grandiflorum* (i.e., “grandiflorum” insects) at five sites: Davy Gooding Bridge (DGB, Qld), Corner of Keevers Drive and Valery Road (KD:VR; NSW), Wyrallah Road and Ingram Road Junction (WR:IR; NSW), Long pocket boat ramp (LPBR; Qld), and Sherlock Road (SR; Qld) (Figure 1, see Table S1 for more details on collection sites). All collections were made under permits issued by the NSW Department of Planning, Industry and Environment (licence number: SL102654), the Director of National Parks, Australian Government (permit number RK737) and the Parks and Wildlife Commission of the Northern Territory (permit number 71037). At each site, we collected up to 30 insects (adults and/or nymphs) of which 15 were measured for proboscis length (i.e., rostrum length) and body length (distance between the distal tip of the head and the anterior tip of the abdomen) using digital callipers (±0.01 mm) before being preserved in 80% ethanol and kept at −80°C until further use for genetic analyses. The remaining insects were used to start laboratory breeding lines for the hybridisation experiments (see below).

For each host plant species, we collected five fruits from five plants at each location (i.e., *n* = 25/site). When fewer than five individual plants were present, 25 fruits were collected from across all individuals found at the site. We measured the size of fruits of all three host plant species using digital callipers (±0.01 mm). The measurements mode differed between the host genera given their different fruit morphologies. For *A. tomentosus* , we measured the fruit size as the width (diameter) and calculated the fruit radius, and for the two *Cardiospermum* species, we measured the distance from the balloon outer capsule to the seed and the seed diameter. These measurements were used to calculate the distance to the centre of the seed for all host plant species, which provides a comparable metric of seed “reachability”.

### 2.2 DArT Library Preparation and Sequencing

The head, thorax, and legs of 166 *Leptocoris tagalicus* insects collected in the field were transferred to 96‐well plates and sent to Diversity Arrays Technology (DArT) for DNA extraction and next generation sequencing. DNA was extracted from the samples using the NucleoMag kit (MACHEREY‐NAGEL). *PstI* and *HpaII* compatible adaptors with two different restriction enzyme overhangs were designed to include the Illumina flowcell attachment sequence, sequencing primer sequence, and “staggered”, varying length barcode region (also see Elshire et al. 2011). The reverse adaptor contained the flowcell attachment region and *HpaII* compatible overhang sequence. Only mixed *PstI*—*HpaII* restriction fragments were PCR amplified using the following conditions: initial denaturation at 94°C for 1 min followed by 30 cycles of 94°C for 20 s, 58°C for 30 s, 72°C for 45 s, and a final extension at 72°C for 7 min.

After PCR amplification, equimolar amounts of amplified PCR product were bulked and subjected to 100 cycles of sequencing (single reads) on the Illumina NovaSeq, NovaSeq X+ sequencer. Sequences generated from each lane were processed using proprietary DArT analytical pipelines. In the initial pipeline, poor quality sequences were removed, with more stringent filtering parameters applied to the barcode region compared to the rest of the sequence, ensuring the reliable assignment of the sequences to specific samples (based on the “barcode split”). Technical replicates were created for ~20% of samples and were carried through the entire sequencing and library preparation process to generate a ‘reproducibility’ score for each locus.

### 2.3 Single Nucleotide Polymorphism (SNP) Filtering

Single Nucleotide Polymorphisms (SNPs) were filtered using the *DartR* R package (Gruber et al. 2018; R Core Team 2023). First, all monomorphic loci were removed, followed by a reproducibility filter of 0.9 and individual call‐rate of 0.7. Individual locus call‐rates were filtered to 0.85, and the depth of coverage was bounded between 8 and 200. Minor allele frequency was restricted to 0.02, after which monomorphic loci were again filtered out. Secondaries (i.e., where more than one SNP per tag was present) were filtered at random. The resulting genotype dataset was then filtered for Hardy–Weinberg Equilibrium at each collection site using a false discovery rate of 0.5 and was corrected for multiple comparisons (all sites had more individuals than the minimum of five used to exclude sites from the analysis, and as such, no insects were excluded). This dataset was then filtered for linkage disequilibrium (LD) using the indep‐pairwise function in PLINK V1.9 (Purcell et al. 2007), for which we used comparisons between all loci and a window size of 1000 kb, with an *R* ^2^ of 0.5.

### 2.4 Genetic Diversity and Structure

We used the filtered genomic dataset to calculate site‐level genetic diversity metrics (observed (*H*o) and unbiased expected heterozygosities (*uHe*) and inbreeding coefficients (*F*_IS_)) and pairwise *F*_ST_ values between all collection sites using the *dartR* R package. We inferred the probable number of genetic clusters using the sparse non‐negative matrix factorisation algorithm of the “snmf” function in the *LEA* R package (Frichot and François 2015), which identifies the optimal number of clusters (*K*) through a minimum cross‐entropy analysis. This analysis was run for *K* values ranging between one and 11, using 10,000 iterations.

### 2.5 Hybridisation Experiment

We conducted common garden experiments to further evaluate genetic differentiation and estimate the heritability of the proboscis length and body size of *L. tagalicus*. Additionally, we assessed the extent of population genetic differentiation between insect biotypes by comparing the performance of parental and hybrid crosses. Live insects collected from each site were kept in a temperature‐controlled glasshouse, covered internally and externally by shade cloth, at Macquarie University's Plant Growth Facility (6:00–10:30 at 26°C, 10:30–16:00 at 28°C, 16:00–18:00 at 26°C, and 18:00–06:00 at 20°C). For our “tomentosus” × “grandiflorum” crosses (breeding experiment 1) we created one breeding line from all insects collected on *A. tomentosus* and one breeding line from “grandiflorum” insects that originated from the DGB site. For our “halicacabum” × “grandiflorum” crosses (breeding experiment 2) we created a “halicacabum” breeding line from insects collected on *C. halicacabum* from all sites. The “grandiflorum” breeding line for this experiment included insects collected from sites KD:VR, WR:IR, LPBR, and SR. These initial breeding lines were maintained for 6 months (i.e., 5–6 generations) before setting up the breeding experiments. Throughout both experiments, insects were fed *C. grandiflorum* seed only, as this host plant fruits all year round. Note that the two breeding experiments form part of separate research projects that differed slightly in some details, as outlined below.

#### 2.5.1 Breeding Experiment 1

We started isolating fifth instars (i.e., last instar stage) of “tomentosus” and “grandiflorum” insects in February 2023, until we obtained at least 20 male and 20 female virgins from each of the two breeding lines. Insects were kept in 90 mm petri dishes lined at the bottom with filter paper. Water was provided in 1.5 mL Eppendorf tubes with a cottonwool plug and changed every 3–4 days. On the 6th of March 2023, isolated adults were randomly paired into four parental (P1) breeding lines: control tomentosus (i.e., ♀ “tomentosus” × ♂ “tomentosus”), two reciprocal hybrid crosses (i.e., ♀ “tomentosus” × ♂ “grandiflorum” and ♀ “grandiflorum” × ♂ “tomentosus”), and control “grandiflorum” (i.e., ♀ “grandiflorum” × ♂ “grandiflorum”). Each P1 breeding line consisted of 10 insect pairs. All paired insects were arranged in a randomised block design consisting of 10 blocks, with each block containing four pairs. Paired insects were placed in 350 mL plastic circular tubs, lined at the bottom with a 90 mm filter paper, with a single cardboard cell from an egg carton for shelter, and with a mesh window in the lid for ventilation. Pairs of insects were fed two halved *C. grandiflorum* seeds every 3–4 days; old food was only removed if observed to be mouldy. Insect pairs were also provided with a 5 mL vial of water (plugged with cottonwool) which was changed weekly. The position of plastic containers was randomised in each block using RANDOM.ORG (Haahr 2023). The position of blocks in the glasshouse was randomised every 7 days.

Eggs laid during the first 2 days following pairing were discarded as *L. tagalicus* females can lay unfertilised eggs. From day 3 onwards, we counted the daily number of eggs laid by each pair for 15 days and transferred these using forceps to a new ‘egg container’ (350 mL plastic circular tubs, lined at the bottom with a 90 mm filter paper, with a bent cardboard rectangle from an egg carton's lid for shelter for subsequent hatchlings, and with a mesh window in the lid for ventilation; these containers were provided with a 5 mL water vial plugged with cottonwool but not food). In pairs where the male died before the female, the female was kept alive to continue laying fertile eggs. At the end of 15 days, we measured proboscis and body length for all P1 adults using digital callipers (±0.01 mm).

One of the first hatchlings to emerge from each P1 pair was moved into a corresponding nymph tracking container (350 mL plastic circular tubs, lined at the bottom with a 90 mm filter paper, with a single cardboard cell from an egg carton for shelter, and with a mesh window in the lid for ventilation; nymphs were fed and watered identically to their parents), while all subsequent hatchlings were moved to a corresponding nymph box (500 mL plastic rectangular tubs, lined at the bottom with paper towel cut to size, with four cardboard cells from an egg carton for shelter, and with a mesh window added in the lid for ventilation; these containers received two 5 mL water vials plugged with cottonwool and half of a *C. grandiflorum* seed for every three nymphs, both of which were changed on the same schedule as parental containers). Nymph boxes contained up to 20 nymphs before a new container was made to prevent overcrowding and competition for resources. The nymph tracking containers were checked daily and the dates at which individuals moulted into different instar stages (5 instar stages in total) and adulthood were recorded. Tracked nymphs that died prior to reaching their 5th instar moult were replaced with a newly hatched nymph on the same day of death where possible or with a nymph from the associated nymph box at first or second instar stage. Any individuals that did not make it to adulthood within 40 days were excluded from the experiment. Once nymphs reached adulthood, they were given 2 days to sclerotise before being measured (proboscis and body length) and sexed. The tracked nymphs were kept alive and used for the next generation where possible.

The number of nymphs in each egg container was counted daily from when the first hatchling emerged until less than five hatchings occurred across all insect pairs in a 2‐day span (after 18 days). Fifth instar nymphs from P1 parents were removed and isolated in 90 mm Petri dishes to be used as virgin parents in the next generation (G1) pairings. One hundred and fifty insects from across all 40 P1 pairs were isolated to ensure enough individuals were available to create 48 G1 pairs. These insects were paired on the 22nd May 2023 using the same design as for the P1 insects. Data collection of all G1 pairs was the same as for P1 pairs.

#### 2.5.2 Breeding Experiment 2

We started isolating 5th instars of “grandiflorum” and “halicacabum” insects in March 2023 until we had obtained at least 30 male and 30 female virgins from each of the two breeding lines. Husbandry of isolated insects followed that for experiment 1. Isolated insects were paired on the 12th of June 2023 into four parental (P1) breeding lines: control “halicacabum” (i.e., ♀ “halicacabum” × ♂ “halicacabum”), two reciprocal hybrid crosses (i.e., ♀ “halicacabum” × ♂ “grandiflorum” and ♀ “grandiflorum” × ♂ “halicacabum”), and control “grandiflorum” (i.e., ♀ “grandiflorum” × ♂ “grandiflorum”). Each P1 breeding line consisted of 15 insect pairs. All paired insects were arranged in a randomised block design consisting of 15 blocks, with each block containing up to eight pairs. The position of containers was randomised in each block using RANDOM.ORG (Haahr 2023). The position of blocks in the glasshouse was randomised every 7 days.

The number of eggs laid by each pair was counted on the third day after pairing (with eggs laid in the first 2 days being discarded before the first count) and then every third and fourth day (alternating) up until the 15th day following pairing. As in experiment 1, in pairs where the male died before the female, the female was kept alive to continue laying fertile eggs. At the end of 15 days, we measured traits of all P1 adults as in experiment 1.

The tracking of nymph development, counting of nymphs, and creation of nymph containers followed the methods described for experiment 1. To prepare for G1 pairings, we isolated up to 10 fifth instar nymphs from each P1 pair and raised them to adulthood. These virgin insects were paired on the 11th of August 2023. Pairing methods for the “grandiflorum” and “halicacabum” lines were generally the same as for P1. For our hybrid lines, we paired eight female offspring of the ♀ “halicacabum” × ♂ “grandiflorum” P1 crosses with eight male offspring of the ♀ “grandiflorum” × ♂ “halicacabum” P1 crosses and eight female offspring of the ♀ “grandiflorum” × ♂ “halicacabum” P1 crosses with eight male offspring of the ♀ “halicacabum” × ♂ “grandiflorum” P1 crosses. Data collection of all G1 pairs was the same as for P1 pairs.

### 2.6 Insect and Host Plant Trait Analyses

Differences in the distance to seed centre for the three host plant species were assessed using a one‐way ANOVA and a Tukey's post hoc analysis. Insect proboscis length and body size were analysed using two‐way ANOVAs with sex and host plant, and their interaction, as explanatory variables. Significant means were separated using Tukey HSD post hoc analyses. These analyses were done in the R statistical environment (R Core Team 2023).

Variance‐to‐mean ratios were computed for fixed effects to assess their dispersion. When modelling egg production, relative fecundity, and development time using generalised linear mixed‐effects models (GLMMs), the random effect of “experiment” had minimal variance (*σ* ^2^: 3.46 × 10^−12^ to 4.55 × 10^−10^; results not shown). We therefore co‐analysed the data from both experiments using generalised linear models (GLMs).

We compared egg production (data overdispersed, see results) between cross types collected from different host plants and reared under common garden conditions by fitting a GLM with a quasipoisson error distribution using the *glm* function in the base R statistical environment. Relative fecundity (data overdispersed) was compared between cross types collected from different host plants and reared under common garden conditions by fitting a GLM that included an offset (log number of nymphs of best performing pair) for estimating the model coefficients, with a quasipoisson error distribution in the base R statistical environment. We compared development time (data underdispersed) using a GLM with a Conway‐Maxwell‐Poisson error distribution using the *glmmTMB* R package (Brooks et al. 2017). Post hoc pairwise comparisons for all GLMs were performed to identify significant differences between the different cross types, using estimated marginal means (EMMs) implemented in the *emmeans* R package (Lenth 2022).

We used separate linear regressions to estimate the correlation between mid‐parent and offspring measurements for proboscis length and body length. We then estimated the narrow sense heritability of these traits with bivariate animal models using the *MCMCglmm* R package (Hadfield 2010). We used a Gaussian model with 100,000 iterations, 100,000 burn‐in, and 10 thinning. We used an inverse‐Wishart prior with V = diag(2)*(0.002/1.002) and nu = 0.002 for both models. We also included sex and cross type (i.e., three control and two hybrid crosses) as fixed effects in the models. We checked all variables for autocorrelation and convergence and used the posterior distribution of variance components to infer mean heritability and a 95% confidence interval for all traits that returned significant pMCMC values (< 0.05). Estimates of narrow sense heritability for both traits were calculated as genetic variance/(genetic variance + residual variance).

## 3 Results

### 3.1 Single Nucleotide Polymorphism (SNP) Filtering

Initial quality filtering of the SNP data resulted in 165 individuals and 11,846 SNPs that were retained for further downstream filtering. At the site‐level, none of the SNPs were identified as violating assumptions of the Hardy–Weinberg Equilibrium. Filtering for linkage disequilibrium resulted in the removal of 7841 loci. We therefore retained a final genetic data set consisting of 165 individuals and 4005 SNPs.

### 3.2 Genetic Diversity and Structure

At site level, observed heterozygosity ranged between 0.195 and 0.225 (average 0.21 ± 0.011), *uH*_E_ between 0.272 and 0.291 (average 0.282 ± 0.008) and *F*_IS_ between 0.226 and 0.282 (average 0.257 ± 0.020) (Table 1). Pairwise *F*_ST_ values ranged between −0.0024 (i.e., between sites BSR and SR) and 0.05 (i.e., between sites KD:VR and DRC). The average pairwise *F*_ST_ between sites was 0.019 (±0.016) (Table 2).

**TABLE 1:** Summary of genetic diversity metrics of *Leptocoris tagalicus* biotypes collected from different sites (average per site and standard deviation).

*L. tagalicus* biotype	Population ID	*H*o	*H*o SD	*uH*_E_	*uH*_E_ SD	*F*_IS_
“halicacabum” insects	EAR	0.198	0.161	0.274	0.179	0.278
	MWK	0.198	0.163	0.272	0.179	0.271
	MFTP	0.195	0.160	0.272	0.180	0.282
	DRC	0.198	0.162	0.273	0.180	0.277
“grandiflorum” insects	LPBR	0.214	0.161	0.283	0.173	0.245
	SR	0.215	0.160	0.286	0.173	0.250
	DGB	0.209	0.157	0.285	0.174	0.268
	WR:IR	0.214	0.159	0.291	0.171	0.263
	KD:VR	0.218	0.167	0.287	0.175	0.241
“tomentosus” insects	SA	0.225	0.161	0.291	0.167	0.226
	BSR	0.223	0.165	0.289	0.171	0.228
	Average	0.210		0.282		0.257

**TABLE 2:** Pairwise *F*_ST_ values between collection sites of different *Leptocoris tagalicus* biotypes.

		“halicacabum” insects				“grandiflorum” insects					“tomentosus” insects	
		EAR	MWK	MFTP	DRC	LPBR	SR	DGB	WR:IR	KD:VR	SA	BSR
“halicacabum” insects	EAR	0										
	MWK	−0.0011										
	MFTP	0.0009	0.0013									
	DRC	0.0015	0.0018	0.0013								
“grandiflorum” insects	LPBR	0.0331	0.0316	0.0355	0.0344							
	SR	0.0271	0.0254	0.0299	0.0280	−0.0016						
	DGB	0.0322	0.0313	0.0332	0.0336	0.0068	0.0001					
	WR:IR	0.0289	0.0273	0.0297	0.0293	0.0035	0.0006	0.0031				
	KD:VR	0.0472	0.0473	0.0499	0.0501	0.0215	0.0159	0.0208	0.0147			
“tomentosus” insects	SA	0.0279	0.0277	0.0307	0.0302	0.0019	−0.0022	0.0032	0.0012	0.0158		
	BSR	0.0274	0.0262	0.0288	0.0295	0.0028	−0.0024	0.0035	−0.0022	0.0151	−0.0020	0

Our admixture analysis identified the most likely number of genetic clusters as two (Figure 1). These two clusters appeared to be strongly related to geography. That is, genomic assignment of “halicacabum” insects collected in the NT corresponded mostly to one genetic cluster while the assignment of “grandiflorum” and “tomentosus” insects from NSW and Qld largely corresponded to the other genetic cluster (Figure 1).

### 3.3 Insect and Host Plant Traits in the Field

**FIGURE 2:**
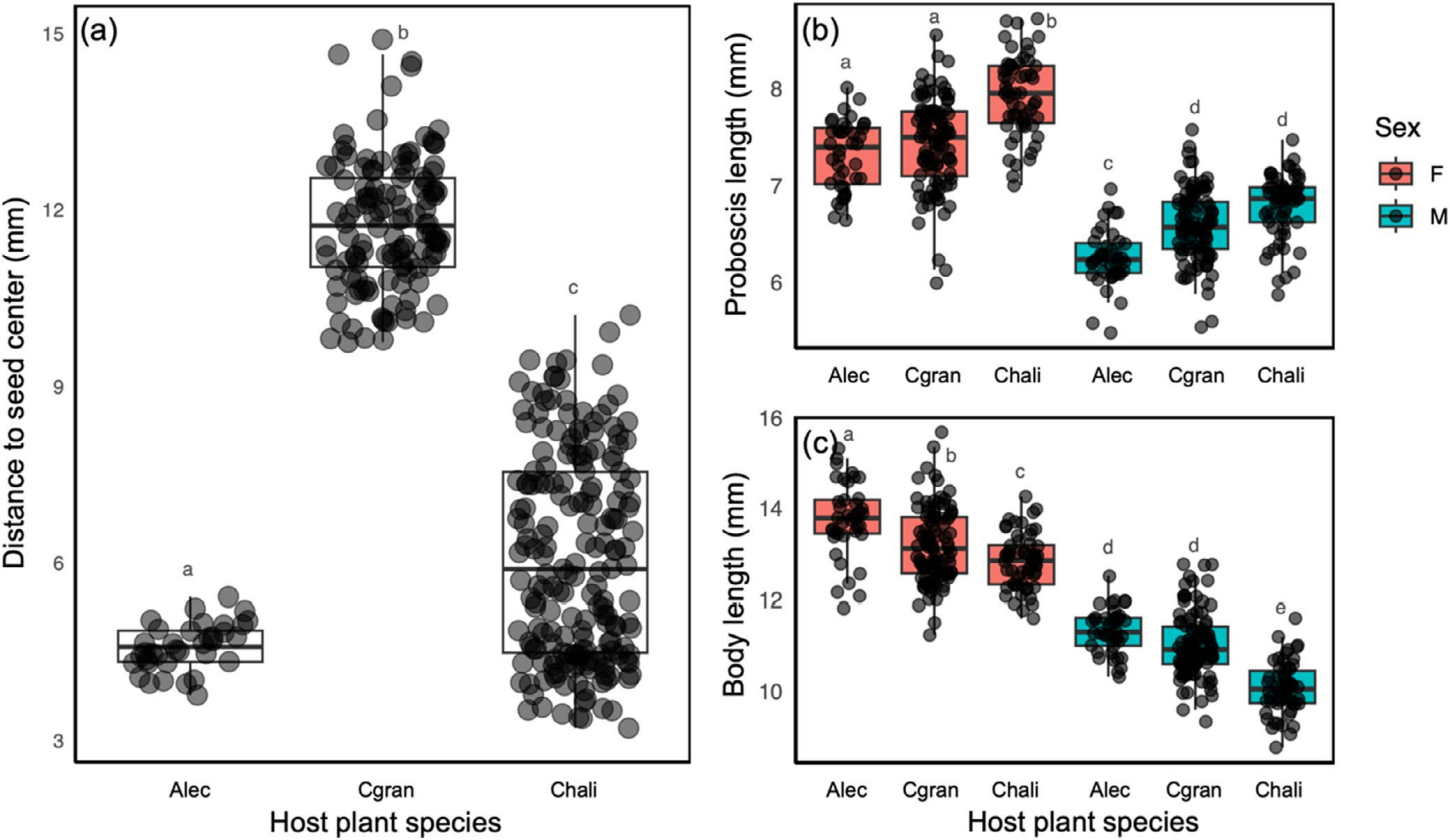
(a) The reachability of seeds in the three study host plant species; *Alectryon tomentosus*, *Cardiospermum grandiflorum* , and *C. halicacabum* . Reachability was calculated as the distance to the seed centre for each species. Significant differences between different host plants are indicated by different letters (*p* < 0.05). (b) The proboscis length of field‐collected male and female *L. tagalicus* insects from the three different host plant species, with significant differences between different host plant × insect sex combinations indicated by different letters above boxplots (*p* < 0.05). (c) The body length of male and female *L. tagalicus* insects collected from three different host plant species, with significant differences between different host plant × insect sex combinations indicated by different letters (*p* < 0.05). Alec, *Alectryon tomentosus*; Cgran, *Cardiospermum grandiflorum* ; Chali, *C. halicacabum* .

The reachability of seeds of the different host plant species, measured as the distance from the outside of the fruit to the centre of the seed, was significantly different (*F*_*2,350*_ = 679.4, *p* < 0.001; Figure 2a). The distance to the seed centre was shortest in *A. tomentosus* (4.59 ± 0.06 mm), followed by *C. halicacabum* (6.1 ± 0.13 mm) and *C. grandiflorum* (11.76 ± 0.09 mm).

A two‐way ANOVA to evaluate the effects of *L. tagalicus* sex and host plant species on insect proboscis length found significant main effects for both sex (*F*_*1,379*_ = 595.51, *p* < 0.001) and host plant species (*F*_*2,379*_ = 53.24, *p* < 0.001), as well as for the interaction between these factors (*F*_*2,379*_ = 5.49, *p* = 0.004; Figure 2b). Tukey's post hoc analyses showed that, irrespective of host plant, female insects always had longer proboscides than male insects. Further, female insects collected from *C. halicacabum* had longer proboscides than those collected from the two other host plant species, while male insects collected from the two *Cardiospermum* species had longer proboscides than those collected from *A. tomentosus* .

A two‐way ANOVA to evaluate the effects of *Leptocoris tagalicus* sex and host plant species on insect body size found significant main effects for sex (*F*_*1, 379*_ = 1125.76, *p* < 0.001) and host species (*F*_*2, 379*_ = 59.81, *p* < 0.001), as well as for the interaction between these two factors (*F*_*2, 379*_ = 6.79, *p* = 0.001; Figure 2c). Tukey's post hoc analyses showed that female insects had larger bodies than male insects. For both sexes, body size varied between host plants, with “tomentosus” insects being the largest, “grandiflorum” insects being intermediate, and “halicacabum” insects being the smallest (Figure 2c).

### 3.4 Hybridisation Experiment

**FIGURE 3:**
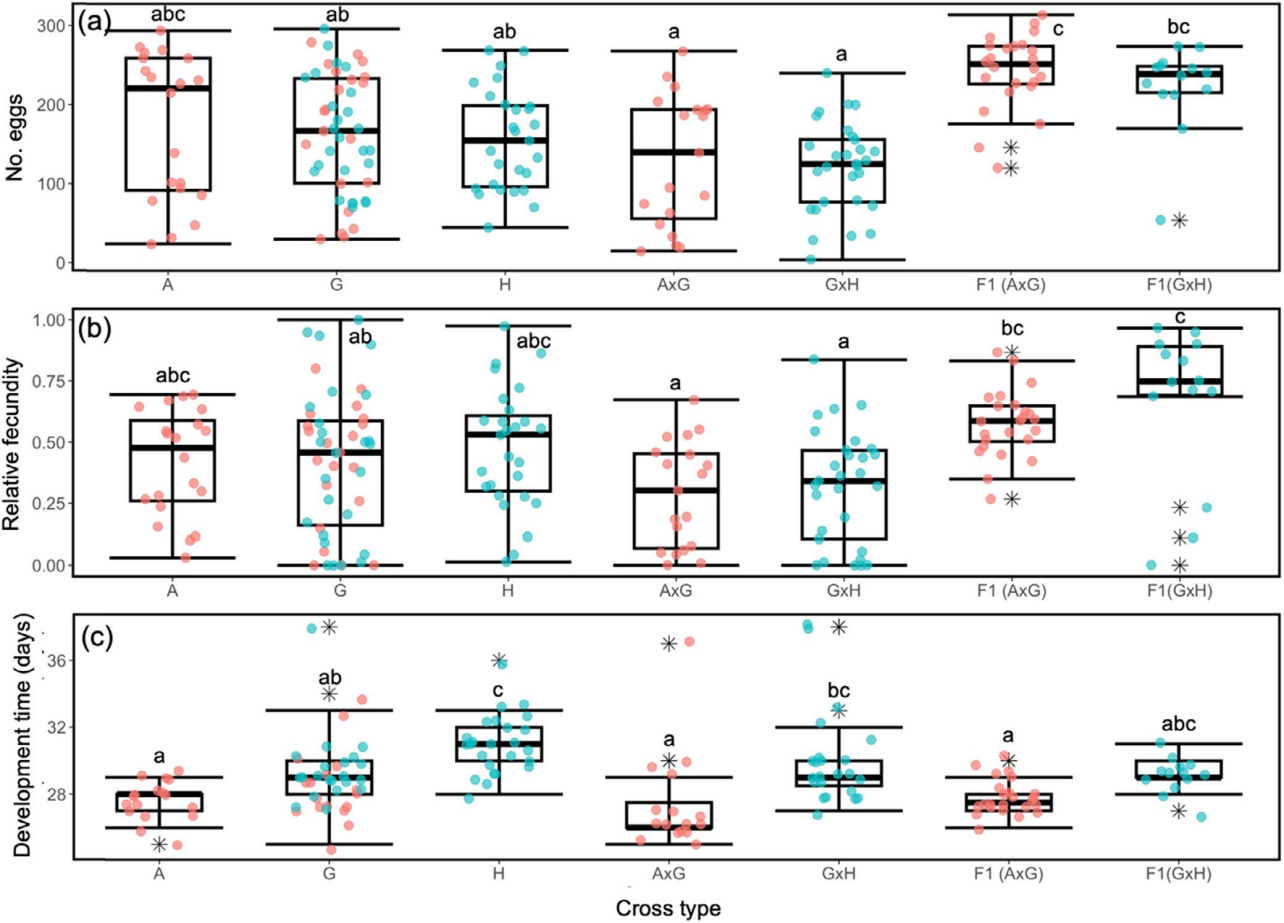
Boxplots of developmental and fitness metrics of *Leptocoris tagalicus* for different cross types fed seeds from *Cardiospermum grandiflorum* : (a) number of eggs laid in 15 days, (b) relative fecundity, (c) number of days taken for tracked nymphs to reach adulthood after hatching. For relative fecundity, each datapoint represents the number of nymphs produced by an insect pair divided by the highest number of nymphs produced by a pair in the hybridisation experiment. Boxes show the interquartile range, the thick bar inside the box is the median, the whiskers represent the highest and lowest non‐outlier data points. Stars show outliers (points outside of 1.5* the interquartile range). Symbol colours represent the breeding experiment: Experiment 1, red; experiment 2, blue. *x*‐axis labels refer to cross types: A, control “tomentosus”; G, control “grandiflorum”; H, control “halicacabum”; A × G, “tomentosus” × “grandiflorum”; G × H, “grandiflorum” × “halicacabum”; F1(A × G), “tomentosus” × “grandiflorum” F_1_; and F1(G × H), “grandiflorum” × “halicacabum” F_1_. Significant differences between cross types (*p* < 0.05) are indicated by lower case letters in all figures.

For egg production, the variance‐to‐mean ratio was 37.03, indicating significant overdispersion in the data. The fitted GLM revealed a significant intercept (estimate = 5.16, SE = ±0.09, *t* = 54.64, *p* < 0.001; Table S2). Insect pairs in the “tomentosus” control cross produced 174 ± 16 eggs on average. The “tomentosus” × “grandiflorum” F_1_ cross pairs produced significantly more eggs compared to pairs in the “tomentosus” control cross (estimate = 242 ± 29 eggs, *t* = 2.80, *p* = 0.005), while the “grandiflorum” × “halicacabum” pairs showed a significant decrease in egg production (estimate = 121 ± 16 eggs, *t* = −2.71, *p* < 0.001). The “tomentosus” × “grandiflorum” and “grandiflorum” × “halicacabum” crosses showed marginal decreases in egg production compared to the “tomentosus” control cross (*p* = 0.05 and 0.07, respectively). The remaining crosses did not show significant differences in egg production. The EMMs post hoc analysis showed that pairs in the “tomentosus” × “grandiflorum” F_1_ cross had the highest egg production, significantly different from all other crosses except the “grandiflorum” × “halicacabum” F_1_ and “tomentosus” control crosses. The remaining crosses produced a similar number of eggs compared to one or more of the other crosses, indicating varying degrees of differences or similarities in their fecundity (Figure 3).

The variance‐to‐mean ratio for the number of hatchlings was 36.8, indicating significant overdispersion in the data. The fitted GLM for relative fecundity had a significantly negative intercept (−0.88 ± 0.132, *t* = −6.695, *p* < 0.0001; Table S3). For insects in the “tomentosus” control cross the mean relative fecundity was 0.41 ± 0.05. Both F_1_ crosses had significantly higher relative fecundity than the “tomentosus” control cross (“grandiflorum” × “halicacabum” F_1_ cross: estimate = 0.67 ± 0.12, *t* = 2.640, *p* < 0.01 and “tomentosus” × “grandiflorum” F_1_ cross: estimate = 0.58 ± 0.1, *t* = 1.958, *p* = 0.05). In contrast, the “tomentosus” × “grandiflorum” had marginally significant lower relative fecundity compared with the “tomentosus” control cross (estimate = 0.29 ± 0.069, *t* = −1.766, *p* = 0.08). The remainder of the crosses had similar relative fecundity to the “tomentosus” control cross. The EMMs post hoc analysis indicated that the two F_1_ crosses had significantly higher relative fecundity than the “grandiflorum” × “halicacabum” and “tomentosus” × “grandiflorum” crosses. The relative fecundity of control crosses was also found to be similar to most crosses except for the “grandiflorum” control cross that had lower relative fecundity than the “grandiflorum” × “halicacabum” F_1_ cross (Figure 3).

For development time, the variance‐to‐mean ratio was 0.19, indicating significant underdispersion in the data. The GLM for development time showed a significant effect of cross type (estimate = 3.31 ± 0.018, *t* = 189.18, *p <* 0.001; Table S4). Compared to the “tomentosus” control cross with a mean development time of 27.5 ± 0.47 days, the “grandiflorum” × “halicacabum” (estimate = 30 ± 0.66 days, *z* = 3.95, *p* < 0.001), “grandiflorum” × “halicacabum” F_1_ (estimate = 29  ± 0.74 days, *z* = 2.07, *p* = 0.03), “grandiflorum” control (estimate = 29 ± 0.59 days, *z* = 2.56, *p* = 0.01) and “halicacabum” control (estimate = 31 ± 0.66 days, *z* = 5.37, *p* < 0.001) crosses had significantly longer development times. Other cross types did not show significant differences. The EMMs showed that the “halicacabum” control cross had the longest average development time, which was statistically similar to the development time of the “grandiflorum” × “halicacabum” and “grandiflorum” × “halicacabum” F_1_ crosses, but significantly longer than all other cross types. The remaining crosses had development times similar to the “grandiflorum” × “halicacabum” F_1_ cross.

**FIGURE 4:**
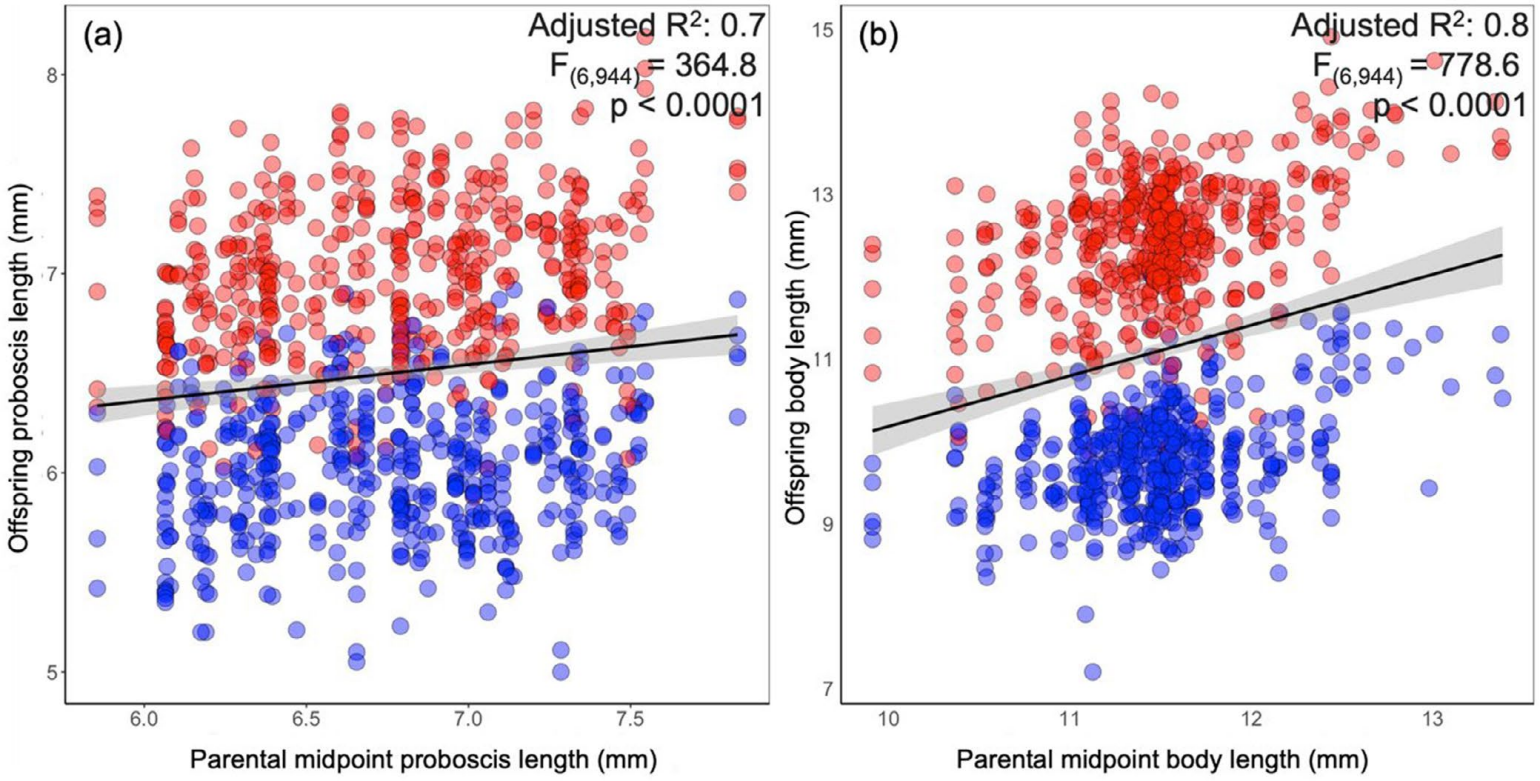
Scatter plots representing mid‐parent and offspring linear regressions for (a) proboscis length and (b) body length. Red‐shaded symbols represent female offspring, and blue‐shaded symbols represent male offspring. The grey shaded areas around the best fit line represent 95% CIs.

Mid‐parent and offspring values for proboscis length and body size were significantly correlated (Figure 4) and our animal models predicted narrow sense heritability to be 0.48 (0.41–0.56; 95% CI) and 0.4 (0.29–0.48; 95% CI) for these two traits, respectively.

## 4 Discussion

We found support for our first hypothesis that insects associated with the two invasive *Cardiospermum* species would be genetically isolated. “Halicacabum” insects from the NT were genetically moderately differentiated from “tomentosus” and “grandiflorum” insects in NSW and Qld (Figure 1). In contrast, the latter two biotypes, which were often collected from proximate locations (within a few kilometres), showed comparatively low genetic differentiation. Our data do not allow us to determine whether the genetic divergence between NT insects and those from NSW and Qld is due to geographic isolation, high host plant fidelity, or both.

The time needed for populations to evolve reproductive isolation remains an important question in evolutionary biology, with some studies suggesting that this can happen quickly (e.g., Schwarz et al. 2005, 2007). We tested whether reproductive isolation is evolving in *L. tagalicus* following its independent colonisation of, and adaptation to, two invasive balloon vine species in Australia. The timing, geography, and demographic dynamics of these two colonisation events by *L. tagalicus* are markedly different. *Cardiospermum halicacabum* was introduced to Australia 200–400 years ago (Le Roux et al. 2025) and now serves as the host for a hybrid biotype of the two *L. tagalicus* subspecies (Andres et al. 2013). On the other hand, *C. grandiflorum* was introduced to the east coast of Australia around a century ago and was subsequently colonised by *L*. *tagalicus* subsp. *tagalicus* (Carroll et al. 2005a).

Given the likely influence of geographic isolation on genetic differentiation in our study, our analyses of reproductive isolation only partially assessed whether the performance (i.e., number of eggs produced, relative fecundity, development time) of “grandiflorum”, “halicacabum”, “tomentosus” insects, and their hybrids, reflects their residence time on different host plants. We did not find support for our hypothesis that F_1_ crosses involving recently versus long‐diverged *L. tagalicus* biotypes will have different fitness responses compared to inter‐biotype and parental control crosses. In both instances, inter‐biotype crosses (i.e., “tomentosus” × “grandiflorum” *and* “grandiflorum” × “halicacabum” crosses) performed similarly to their respective parental control crosses (Figure 3), while both F_1_ crosses had higher performance levels (i.e., egg production and relative fecundity) compared to the inter‐biotype crosses. It is worth noting the slower development of “halicacabum” control insects and the nymphs produced by other crosses involving “halicacabum” insects, which could be due to these insects being fed seeds of *C. grandiflorum* , a host diet that they do not encounter in the NT. We also note that our fecundity data (i.e., number of eggs laid and hatchling emergence) were overdispersed, suggesting additional sources of variation in these data that were not accounted for in our models, such as developmental plasticity or genetic attributes (e.g., inbreeding over successive generations in the laboratory).

Experimental hybridisation is a powerful approach to investigate the magnitude and genetic architecture of population differentiation (Galloway and Etterson 2005). For instance, genetic drift can lead to the fixation of alternate alleles across populations. When these populations hybridise, hybrid vigour (i.e., heterosis) may arise if loci exhibit overdominance or if fixed alleles in different populations are mildly deleterious and recessive. This increase in performance is typically greatest in F_1_ hybrids (Lynch and Walsh 1998), as was the case in our study. Our observation of vigour in biotype F_1_ crosses raises an important question: if these crosses have higher fitness than the parental biotypes, how does trait differentiation persist in *L. tagalicus* populations in the wild? It is possible that the fecundity we measured under laboratory conditions may be obscured by other attributes of diet quality in the wild. For example, in the North American soapberry bug, *Jadera haematoloma* , host quality appears to influence traits like proboscis length through phenotypic plasticity. When different biotypes of this species were reared on either native or non‐native host plants, developmental changes resulted in proboscides that were poorly matched in length to the size of the rearing host's fruit (Cenzer 2017). This “maladaptive plasticity” was most pronounced in individual insects collected from areas where native and invasive hosts coexist, suggesting that ongoing gene flow in these in the field may promote maladaptive trait expression (Cenzer 2017). In our study, we reared *L. tagalicus* on invasive *C. grandiflorum* only. Future research should incorporate multiple host diets to explore how host quality influences trait plasticity in this species.

The persistence of host‐specific insect trait differences under common garden conditions across multiple generations (Figure 2; also see Geraghty et al. 2025), along with our estimates of trait heritability and performance differences between inter‐biotype and F_1_ crosses, provides evidence for differentiation among all studied *L. tagalicus* biotypes. Notably, these differences were observed in both geographically and genetically more isolated (i.e., “halicacabum” and “grandiflorum” insects; *F*_*ST*_ = 0.033) and proximate (i.e., “tomentosus” and “grandiflorum” insects; *F*_*ST*_ = 0.008) biotypes. While ongoing gene flow between “grandiflorum” and “tomentosus” insects may slow the evolutionary adaptation of “grandiflorum” insects to their new host plant, it is not preventing adaptation altogether (Carroll et al. 2005a; Figure 2). Although we did not have the opportunity to sample *L. tagalicus* insects on nearby small‐fruited native host plants such as *Atalaya hemiglauca* in the NT, it is likely that gene flow also occurs between them and “halicacabum” insects (Andres et al. 2013). Previous studies have shown that trait differences can persist between populations despite high gene flow (e.g., Comeault et al. 2015; Yadav et al. 2019; Kardum Hjort et al. 2024). In addition to the role of maladaptive plasticity mentioned above, *L. tagalicus* individuals with short proboscides may also experience lower fitness on *Cardiospermum* host plants compared to those with long proboscides. Conversely, while the performance of insects with long proboscides on smaller‐fruited native hosts will likely be suboptimal (Carroll et al. 1997; Kunte 2007), they will not be physically excluded from feeding. This asymmetry could enable long‐proboscis individuals to mediate gene flow between host plant species, even as trait divergence continues. Lastly, it is also possible that selection is acting on a few genes of major effect to drive differentiation between *L. tagalicus* biotypes (see Riesch et al. 2017), as has been suggested for biotypes of the North American soapberry bug, *J. haematoloma* (Carroll et al. 1997).

Our findings contribute to a growing body of evidence showing that invasive species not only impact native biodiversity ecologically, such as by outcompeting native species, but that they can also drive rapid evolutionary responses in native populations. These impacts may hold significant long‐term consequences for the demographic dynamics and future evolutionary trajectories of both native and invasive species. For instance, along Australia's east coast, Geraghty et al. (2025) found that the recent convergence of the northern (near Brisbane) and southern (near Sydney) *C. grandiflorum* invasion fronts has likely disrupted historical dispersal barriers in *L. tagalicus*, leading to the genetic admixture of previously distinct insect populations. As *L. tagalicus* continues to evolve adaptations to exploit invasive *Cardiospermum* species, it may eventually slow their spread or even contribute to their biological control.

## Author Contributions

**Johannes J. Le Roux:** conceptualization (lead), formal analysis (lead), funding acquisition (lead), investigation (lead), methodology (supporting), project administration (lead), writing – original draft (lead), writing – review and editing (lead). **Levi Brown:** conceptualization (equal), data curation (equal), investigation (equal), methodology (equal), writing – review and editing (equal). **Scott P. Carroll:** conceptualization (equal), funding acquisition (equal), project administration (equal), writing – review and editing (equal). **Jessica A. O'Hare:** formal analysis (equal), writing – review and editing (equal). **Jess M. Herbert:** investigation (equal), methodology (equal), writing – review and editing (equal). **Niah M. Delamotte:** investigation (equal), writing – review and editing (equal). **Nicholas Bersee:** investigation (equal), writing – review and editing (equal). **Sigrid Iredell:** investigation (equal), writing – review and editing (equal). **Rowan M. Clarke:** methodology (equal), writing – review and editing (equal). **Selina Kosak:** investigation (equal), writing – review and editing (equal). **Rachael Y. Dudaniec:** funding acquisition (equal), writing – review and editing (equal). **Dylan M. Geraghty:** conceptualization (equal), formal analysis (equal), investigation (equal), methodology (equal), writing – review and editing (equal).

## Conflicts of Interest

The authors declare no conflicts of interest.

## Supporting information


Appendix S1.

## Data Availability

Genotype (insect SNP) as well as plant and insect morphological data are available in the Zenodo online repository (https://zenodo.org/records/15054308).
