# Supplementary material for: Fitness and Morphology Support Genetic Differentiation Across Different Geographic Scales in a Native Insect Utilising Native vs. Invasive Host Plants
Source: Ecol Evol. 2025 May 12;15(5):e71373. doi: 10.1002/ece3.71373 (PMC12069220; doi:10.1002/ece3.71373)
Supplement: Supplementary file 1 — Appendix S1. [file ECE3-15-e71373-s001.docx]

**Table S1. Details of localities from where *Leptocoris tagalicus* individuals were collected for this study.**

| **Host plant** | **Site** | **State/territory** | **Lat** | **Long** | **Experiment** |
| --- | --- | --- | --- | --- | --- |
| *A. tomentosus* | BSR | Queensland | -27.74 | 153.18 | 1 |
| *A. tomentosus* | SA | Queensland | -27.53 | 152.98 | 1 |
| *C. grandiflorum* | DGB | Queensland | -27.71 | 153.19 | 1 |
| *C. grandiflorum* | WR:IR | New South Wales | -28.88 | 153.30 | 2 |
| *C. grandiflorum* | KD:VR | New South Wales | -30.44 | 153.01 | 2 |
| *C. grandiflorum* | LPBR | Queensland | -27.52 | 153.01 | 2 |
| *C. halicacabum* | DLC | Northern Territory | -13.77 | 130.71 | 2 |
| *C. halicacabum* | MWK | Northern Territory | -12.65 | 132.57 | 2 |
| *C. halicacabum* | EAR | Northern Territory | -12.43 | 132.97 | 2 |
| *C. halicacabum* | MFTP | Northern Territory | -13.75 | 130.70 | 2 |
| *C. grandiflorum* | SR | Queensland | -27.55 | 152.73 | 2 |

**Table S2. Summary of GLM for *Leptocoris tagalicus* egg production.**

|  | **Estimate** | **SE** | **t value** | **Pr(>\|t\|)** |
| --- | --- | --- | --- | --- |
| Intercept | 5.157905 | 0.094405 | 54.63612 | 4.8E-111 |
| crossTypeAG_inter | -0.28552 | 0.146266 | -1.95209 | 0.05 |
| crossTypeAG_F1 | 0.334299 | 0.119284 | 2.802541 | 0.005 |
| crossTypeCH_inter | -0.36041 | 0.133138 | -2.70702 | 0.007 |
| crossTypeCH_F1 | 0.248305 | 0.137276 | 1.808808 | 0.07 |
| crossTypegrandiflorum_control | -0.06285 | 0.113801 | -0.55228 | 0.58 |
| crossTypehalicacabum_control | -0.1071 | 0.127515 | -0.8399 | 0.4 |

**Table S3. Summary of GLM for *Leptocoris tagalicus* relative fecundity.**

|  | **Estimate** | **SE** | **t value** | **Pr(>\|t\|)** |
| --- | --- | --- | --- | --- |
| Intercept | -0.88083 | 0.131564 | -6.69506 | 2.89E-10 |
| crossTypeAG_inter | -0.3691 | 0.208957 | -1.76641 | 0.08 |
| crossTypeAG_F1 | 0.326005 | 0.166495 | 1.958051 | 0.05 |
| crossTypeCH_inter | -0.2572 | 0.180963 | -1.42126 | 0.15 |
| crossTypeCH_F1 | 0.477055 | 0.180707 | 2.639938 | 0.009 |
| crossTypegrandiflorum_control | 0.004625 | 0.156973 | 0.029465 | 0.98 |
| crossTypehalicacabum_control | 0.141282 | 0.168645 | 0.837748 | 0.4 |

**Table S4. Summary of GLM for *Leptocoris tagalicus* development time.**

|  | **Estimate** | **SE** | **t value** | **PR(>\|t\|)** |
| --- | --- | --- | --- | --- |
| Intercept | 3.317389 | 0.016937 | 195.87 | 2E-16 |
| crossTypeAG_F1 | -0.00547 | 0.024358 | -0.22 | 0.8 |
| crossTypealectryon_control | 0.005846 | 0.02211 | 0.26 | 0.7 |
| crossTypeCH_inter | 0.086702 | 0.021932 | 3.95 | 7.71E-05 |
| crossTypeCH_F1 | 0.052555 | 0.025347 | 2.07 | 0.03 |
| crossTypegrandiflorum_control | 0.05172 | 0.020214 | 2.56 | 0.01 |
| crossTypehalicacabum_control | 0.115307 | 0.021456 | 5.37 | 7.70E-08 |
